# Adsorptive removal of fluoride from water using hydroxyapatite synthesized from marine shell waste

**DOI:** 10.1038/s41598-025-11132-5

**Published:** 2025-07-15

**Authors:** Shantanu Singh, Gokulakrishnan Murugesan, Ramesh Vinayagam, Thivaharan Varadavenkatesan, Raja Selvaraj

**Affiliations:** 1 Department of Chemical Engineering, Manipal Institute of Technology, Manipal Academy of Higher Education, Manipal, Karnataka 576104 India; 2 Department of Biotechnology, M.S.Ramaiah Institute of Technology, Bengaluru, Karnataka 560054 India; 3 Department of Biotechnology, Manipal Institute of Technology, Manipal Academy of Higher Education, Manipal, Karnataka 576104 India

**Keywords:** Adsorption, *Marcia recens* shells, Hydroxyapatite, Fluoride removal, Spiking studies, Fluoride adsorption mechanism, Environmental sciences, Chemical engineering, Nanoscale materials, Chemical engineering, Environmental chemistry, Green chemistry, Materials chemistry

## Abstract

Fluoride contamination in groundwater threatens human health and ecological systems, necessitating cost-effective and efficient remediation strategies. This study synthesized hydroxyapatite (MRS-HAp) from *Marcia recens* shells through chemical precipitation to serve as a potential adsorbent for the removal of fluoride. The prepared MRS-HAp exhibited a specific surface area of 100.42 m^2^/g. FESEM analysis revealed an irregular, closely packed structure with a mean diameter of 28.89 nm. EDS determined a Ca/P molar ratio of 1.6, while XRD analysis confirmed a hexagonal crystalline lattice with a crystallite diameter of 32.18 nm. XPS identified a fluoride peak at 684.58 eV, confirming adsorption. The adsorption dataset obeyed pseudo-second-order kinetics and Langmuir isotherm, pointing to chemisorption and monolayer coverage, with a maximum adsorption capacity of 19.19 mg/g. Spiked water experiments demonstrated robust fluoride removal efficiencies across diverse real-world water matrices. MRS-HAp showed reasonable regeneration potential for fluoride removal, retaining significant adsorption capacity over four cycles. These results position MRS-HAp as a cost-effective and sustainable adsorbent for fluoride removal in water treatment applications.

## Introduction

Various emerging pollutants, including pesticides, fertilizers, and heavy metals like mercury, lead, cadmium, and arsenic, along with non-metallic contaminants like nitrates, phosphates, and fluoride, pose significant risks to water quality^1^. Among these, fluoride is vital for human well-being, yet its concentration must be carefully regulated to avoid harmful consequences. Fluoride is a geogenic contaminant naturally present in rocks, soil, and volcanic emissions. It leaches into groundwater through weathering, mineral dissolution, and geothermal activities, leading to its accumulation in water sources. Additionally, anthropogenic activities such as aluminum production, coal burning, and various industrial processes contribute to fluoride pollution^2^. The World Health Organization (WHO) advises maintaining fluoride levels in drinking water between 0.5 and 1.5 mg/L is ideal. Concentrations below 0.5 mg/L can cause dental caries, while prolonged exposure to more than 1.5 mg/L can severely impact health, and cause dental fluorosis and skeletal fluorosis^3^. Epidemiological studies also link fluoride toxicity to neurological effects, including reduced intelligence in children, and potential reproductive health concerns. Given the serious health risks associated with fluoride contamination, the development of effective and affordable defluoridation technologies is crucial.

There are several treatment methods for fluoride removal, including ion exchange, precipitation, coagulation, and membrane processes. Amongst these, adsorption is a cost-efficient and energy-efficient option^4^. It is known to be facile, requiring less operational complexity compared to traditional methods, which are prone to fouling and demand frequent maintenance^5^. Additionally, adsorption is more selective, targeting specific contaminants at low concentrations, and does not generate sludge^6^.

Various materials were studied for fluoride adsorption, including activated carbon, biochar, magnetic biochar, chitosan, metal hydroxides, metal-organic frameworks, and layered double hydroxides^7^. While some of these adsorbents exhibit high efficiency, they often involve significant energy consumption, high operational costs, and potential toxicity risks if ingested. In contrast, hydroxyapatite (HAp) presents a more sustainable alternative because of its low cost, biocompatibility, simple process, and minimal energy requirements, making it particularly suitable for use in developing and low-income regions^8^. HAp is a biocompatible calcium phosphate compound with the chemical formula Ca_10_(PO_4_)_6_(OH)_2_. It closely matches the mineral composition of natural sources like eggshell, seashell, bone, and dental enamel, making it an effective and safe adsorbent for fluoride removal^7^.

HAp can be prepared by many methods, including mechanochemical synthesis, precipitation, hydrothermal treatment, thermal calcination, and sol-gel processing^9^. Among these, chemical precipitation is the most straightforward, cost-effective, and scalable approach, as it ensures high purity at low temperatures. In contrast, hydrothermal and sol-gel methods are expensive, time-consuming, and complex^10^. Sustainable HAp synthesis can incorporate biogenic waste materials such as shells and bones, aligning with green chemistry principles while reducing environmental impact and addressing waste management challenges. Additionally, this approach lowers production costs, making HAp even more viable for fluoride removal applications^11^.

This study focuses on developing a cost-effective HAp adsorbent from *Marcia recens* shells using the precipitation method. The synthesized HAp was comprehensively assessed in terms of its formation process, structural properties, adsorption performance, and suitability for long-term water treatment. The synthesized HAp was characterized using many techniques. The adsorption experiments were conducted to optimize fluoride removal conditions, and the results were validated through spiking studies. The findings highlight the effectiveness of this sustainable HAp adsorbent.

## Materials and methodology

### Materials used

Orthophosphoric acid (85%), ammonia liquid (25%), sodium hydroxide pellets, and sodium fluoride were bought from Merck, India. *Marcia recens* shells (MRS) were collected from the local fish market, Udupi, Karnataka.

### Synthesis

**Fig. 1:**
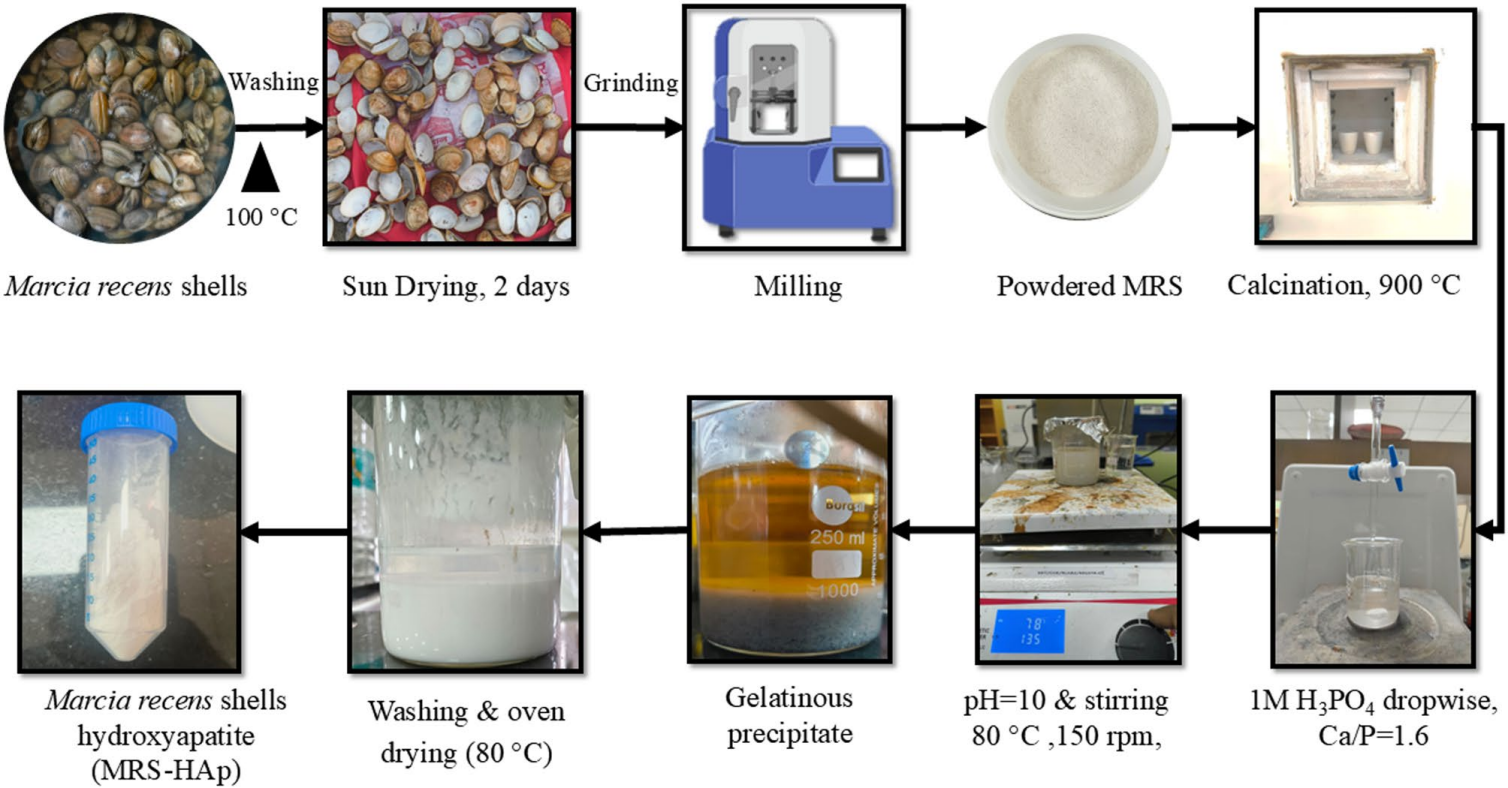
Schematic representation of the synthesis process of hydroxyapatite from *Marcia recens* shells (MRS-HAp).

The collected shells were washed with water and boiled to remove membranes (Fig. 1). The cleaned shells were kept for sun drying for 2 d and were subsequently powdered in a ball mill and sieved. Further, the powder was calcined at 900 °C, for 2 h in a muffle furnace to obtain CaO. The contents were mixed with distilled water and stirred at 200 rpm for 120 min to obtain Ca(OH)_2_. The resultant solution was kept idle for 24 h and subsequently, 100 mL of H_3_PO_4_ was added dropwise into this solution to maintain the Ca/P ratio of 1.6. pH was maintained at 10 with NH_4_OH. This solution was stirred at 80 °C at 150 rpm for 2 h until a white gelatinous precipitate was formed. After cooling to room temperature, the precipitate was decanted and subsequently washed three times with deionized water to achieve a neutral pH. The neutralized product was then dried in a hot air oven at 80 °C. The resulting precipitate was labeled as *Marcia recens* shells hydroxyapatite (MRS-HAp).

### Characterization

The crystallinity and phase structure of MRS-HAp were analyzed with X-ray diffractometer (XRD, D8 Advance, Bruker, Germany). A Field Emission Scanning Electron Microscope (FESEM, Zeiss Sigma 300, Carl Zeiss, Germany), equipped with Energy-Dispersive X-ray Spectroscopy (EDS, Oxford Instruments, UK), was used to examine the surface topography and elemental compositions. The functional groups were identified using a Fourier Transform Infrared spectrophotometer (FTIR, Shimadzu 8400 S, Japan). The pore volume and specific surface area (SSA) were evaluated with the Brunauer-Emmett-Teller (BET) technique (Smart Instrument, Mumbai). The oxidation states and elemental properties of the produced MRS-HAp were assessed through an X-ray photoelectron spectroscope (XPS), Thermo Fisher Scientific, UK.

### Adsorption experiments

100 mg/L fluoride stock solution was prepared as per Indian standard code IS 3025-60 (2016)^12^. The pH effect (3–11) was studied by mixing 0.5 g/L MRS-HAp in 100 mL of 10 mg/L fluoride solution agitated at 150 rpm, 12 h. The effect of dosage was carried out by introducing MRS-HAp dosages between 0.3 and 1.0 g/L in 10 mg/L fluoride solution at optimized conditions. Subsequently, the impacts of initial concentration and adsorption time were studied by adding the optimized dosage of MRS-HAp in fluoride concentration of 5 to 25 mg/L at optimized pH. Further, the effect of temperature was investigated at optimal pH, dosage, concentration, and contact time at temperatures between 293 and 313 K. After the adsorption experiments, 1.5 mL of the sample was withdrawn and centrifuged at 10,000 rpm for 10 min to separate the spent adsorbent. The residual fluoride concentration was analyzed using a fluorimeter (Thermo Scientific, Orion ISE Fluorimeter, USA). Triplicates of each study were performed, and the percentage removal (R) and adsorption capacity (q_e_) were determined using the Eqs. (1) and (2).

1 $${\text{R~}}=\frac{{{{\text{C}}_0} - {{\text{C}}_{\text{f}}}}}{{{{\text{C}}_0}}} \times 100$$

2 $${{\text{q}}_{\text{e}}}=\frac{{{{\text{C}}_0} - {{\text{C}}_{\text{e}}}}}{{\text{W}}}{\text{~}} \times {\text{~V}}$$

wherein C_0_, C_f,_ and C_e_ denote respective initial, final, and equilibrium fluoride concentration, V: volume of solution, and W: weight of MRS-HAp added.

### Adsorption modeling

The adsorption isotherm investigations were done to analyze the interaction of the adsorbent and adsorbate. This was achieved by fitting the dataset to the non-linear Langmuir, Freundlich, and Temkin isotherms model equation. Kinetic studies were performed by fitting the data set to pseudo-first-order (PFO), pseudo-second-order (PSO), and intraparticle diffusion (IPD) models. The thermodynamic factors including standard entropy change (ΔS°), Gibbs free energy (ΔG°), and enthalpy change (ΔH°) were studied to understand the spontaneity of the adsorption process. Van’t Hoff equation was employed to calculate these parameters. To evaluate the goodness of fit of the models, the regression coefficient (R^2^) and chi-squared (χ^2^) values were compared. R^2^ indicates how well the model explains the variability of the experimental data, with values closer to 1 denoting a stronger correlation and better fit. The χ^2^ value reflects the degree of agreement between the experimental data and the adsorption model, with lower χ^2^ values indicating a better fit and higher values suggesting a poor fit. All the respective equations are represented in Table 1.

**Table 1:** Parameters of kinetic, isotherm, and thermodynamic models for the adsorption of fluoride onto MRS-HAp.

Model	Equation		Parameter
Isotherm models			
Langmuir	$\:{\text{q}}_{\text{e}}\:=\:\frac{{\text{q}}_{\text{m}}\text{b}{\text{C}}_{\text{e}}}{(1\:+\:\text{b}{\text{C}}_{\text{e}}\:)}$		$\:{q}_{m}$ (mg/g) = 19.19 b (L/mg) = 3.12 R^2^ = 0.9626 χ^2^ = 2.65
Freundlich	$\:{\text{q}}_{\text{e}}=\:{K}_{f}\left[{\text{C}}_{\text{e}}^{\left(\frac{1}{\text{n}}\right)}\right]$		K_f_ (L/mg) = 13.82 *n* = 8.24 R^2^ = 0.9077 χ^2^ = 6.55
Temkin	$\:{\text{q}}_{\text{e}}\:=\:\text{B}.\text{l}\text{n}\left(\text{A}{\text{C}}_{e}\right)$		A (L/mg) = 827.45 B (J/mol) = 2.04 R^2^ = 0.6313 χ^2^ = 7.80
Kinetic models			
Pseudo first order	$\:{{\text{q}}_{\text{e}}=\text{q}}_{\text{t}}[1-{\text{e}}^{-\:{k}_{1}\text{t}}]$		q_e_ (mg/g) = 7.71 k_1_ (1/min) = 0.35 R^2^ = 0.9697 χ^2^ = 0.23
Pseudo second order	$\:{\text{q}}_{\text{t}}=\frac{{\text{k}}_{2}{\text{q}}_{\text{e}}^{2}\text{t}}{1+{\text{q}}_{\text{e}}{\text{k}}_{2}\text{t}}$		q_e_ (mg/g) = 8.00 k_2_ (g/mg.min) = 0.083 R^2^ = 0.9873 χ^2^ = 0.09
Intraparticle diffusion	$\:{\text{q}}_{\text{t}}={K}_{d}{\text{t}}^{0.5}+\text{C}$		K_d_ (mg/g.min^0.5^) = 0.44 C (mg/g) = 3.71 R^2^ = 0.5508 χ^2^ = 3.43
Thermodynamic model			
Van’t Hoff Model	$\:\text{ln}{K}_{t}=\left[\frac{{\Delta\:}\text{S}^\circ\:}{R}-\frac{{\Delta\:}\text{H}^\circ\:}{RT}\right]$	Temperature (K)	293 298 303 308 313
		Δ G°, kJ/mol	− 2.70 − 2.94 − 4.08 − 5.27 − 5.69
		Δ H°, kJ/mol	45.57
		Δ S°, J/mol K	164.17
		R^2^	0.9556
		χ^2^	0.42

q_e_: Equilibrium adsorption capacity; C_e_: equilibrium fluoride concentration; K_d_: IPD rate constant; C: IPD intercept; q_m_: Monolayer adsorption capacity; b: Langmuir constant; K_f_: Freundlich constant; 1/n: adsorption intensity; k_1_: PFO constant; k_2_: PSO constant; ΔG°: (= – RT ln K_t_), standard Gibbs free energy; K_t_: (q_e_/C_e_), distribution factor; ΔH°: standard enthalpy change and ΔS°: standard entropy change; B & A: Temkin constants.

Significant values are in bold.

### Spiking studies in real water samples

The behavior of fluoride in complex water matrices can be better understood through spiked or simulated water studies. In this study, a 10 mg/L fluoride solution was prepared by spiking real water samples collected from diverse places such as Manipal Lake (MLW), Arbi Falls (AFW), the Swarna River (SRW), and groundwater (GW) from a well. The real water samples were collected using the grab sampling method, and adsorption analysis was performed following the optimized conditions.

### Regeneration and reusability studies of MRS-HAp

Regeneration studies of MRS-HAp were carried out to evaluate its reusability. 3 M NaOH was chosen as the eluent because fluoride adsorption in an acidic medium is governed by the protonation of fluoride ions, whereas desorption requires their deprotonation. Since the solution pH is lower than the point of zero charge (pH < pH_zpc_), an eluent with a pH greater than pH_zpc_ is necessary to initiate deprotonation^13^. The adsorption experiment was conducted using an initial fluoride concentration of 10 mg/L, an adsorbent dosage of 0.50 g/L, an initial pH of 3, an agitation speed of 150 rpm, a contact time of 12 h, and a temperature of 303 K. Further, the spent MRS-HAp was separated using Whatman filter paper and thoroughly washed with distilled water until pH 7 was achieved. The spent MRS-HAp was then dried overnight in an oven at 105 °C. Furthermore, the regeneration process was initiated by soaking the dried MRS-HAp in 3 M NaOH for 17 h, followed by separation using a Whatman filter paper and thorough washing with distilled water until pH 7 was attained^14^.

## Results and discussions

### Characterization

#### Morphological features of MRS-HAp

**Fig. 2:**
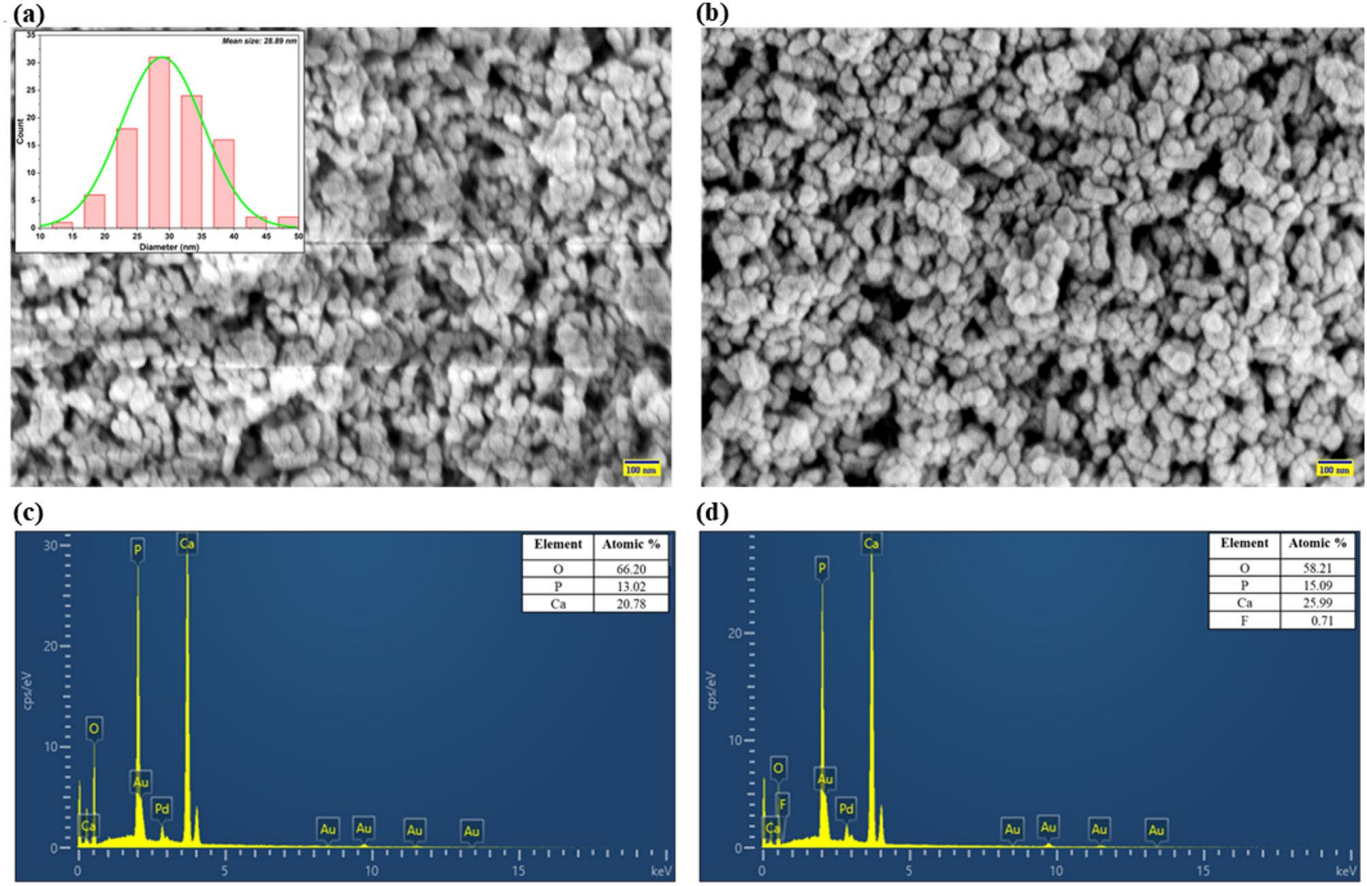
FESEM images of MRS-HAp (**a**) before and (**b**) after fluoride adsorption; EDX spectra (**c**) before and (**d**) after fluoride adsorption.

The FESEM analysis of MRS-HAp was conducted before and after fluoride adsorption (Fig. 2a, b). Before adsorption, FESEM revealed non-homogeneous, irregularly shaped, and tightly packed particles, suggesting strong aggregation of MRS-HAp particles (Fig. 2a), consistent with Zou et al. findings using mussel shells for HAp synthesis^15^. The mean diameter of MRS-HAp was 28.89 nm. The lack of a stabilizing agent during synthesis explains the variability in particle shape and size. After adsorption, the FESEM image (Fig. 2b) showed a smoother structure, indicating successful fluoride adsorption.

The EDS analysis of MRS-HAp was conducted on samples before and after fluoride adsorption (Fig. 2c, d). Before adsorption, the primary elements detected were Ca, O, and P. The Ca/P ratio was 1.60, closely matching the theoretical value of 1.67 and aligning with the literature^16^. After adsorption, EDS showed a significant increase in signal intensity, with the atomic weight percentages of Ca rising from 20.78 to 25.99% and P from 13.02 to 15.09%. Fluoride was detected at 0.71% atomic weight, indicating its successful adsorption onto MRS-HAp (Fig. 2d). These changes, along with a decrease in O intensity due to OH⁻ replacement by fluoride ions, suggest the formation of fluorapatite and structural modifications in the HAp lattice. The fluoride uptake aligns with the literature value of 0.91% for chemically synthesized HAp^17^. The shifts in elemental intensities reflect surface interactions and chemical bonding between MRS-HAp and fluoride, corroborating trends observed by Zou et al. with mussel shells^15^, and Scheverin et al. with sunflower husk-derived HAp for fluoride removal^18^.

The specific surface area (SSA) and total pore volume (PV) of MRS-HAp were measured at 100.42 m^2^/g and 0.21 cm^3^/g, respectively, with a mean pore diameter of 8.36 nm, confirming its mesoporous surface morphology. The SSA of MRS-HAp surpasses values from recent studies, including mussel shells (35.3 m^2^/g)^15^, and cattle bone char (79.34 m^2^/g)^19^. With a significantly higher SSA, MRS-HAp stands out as a highly promising adsorbent compared to these materials.

#### XRD analysis

**Fig. 3:**
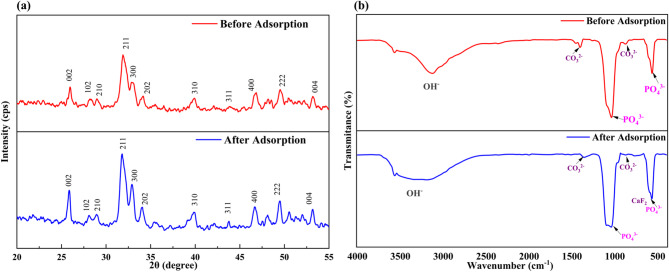
Structural and functional group analysis of MRS-HAp (**a**) XRD pattern and (**b**) FTIR spectrum.

The XRD diffraction patterns (Fig. 3a) exhibit prominent peaks at 25.96°, 28.23°, 29.01°, 31.90°, 32.96°, 34.05°, 39.85°, 46.74°, 49.52°, and 53.20°, corresponding to the Miller indices (002), (102), (210), (211), (300), (202), (310), (400), (222), and (004), respectively. These peaks match the hexagonal lattice structure of MRS-HAp, consistent with JCPDS file No. 09-0432^20^. Comparable diffraction patterns have been observed in HAp derived from, mussel shells^15^, and cattle bone char^19^. The lattice parameters of MRS-HAp were calculated as ‘a’ = 9.17 Å and ‘c’ = 6.85 Å, closely approximating the standard values of 9.41 Å and 6.88 Å, confirming its hexagonal phase. The crystallite diameter was calculated using the Debye-Scherrer equation for the most prominent signal at 31.90° (211 plane) as 32.18 nm.

#### FTIR results

The functional groups involved in fluoride adsorption by MRS-HAp were examined using FTIR (Fig. 3b). The spectra display distinct vibrational features linked to key functional groups. Broad peaks between 3200 and 3600 cm^−1^ indicate O–H stretching, while the band at 620–780 cm^−1^ corresponds to the OH liberation mode^21^. Characteristic CO_3_^2−^ vibrations at 1400–1500 cm^−1^ suggest minor impurities in HAp^22^. Peaks in the 1000–1100 cm^−1^ range align with PO_4_^3−^ groups, consistent with Shahid et al.’s findings on bone char-derived HAp^19^. The signals at 962, and 602, 572 cm^−1^ are attributed to phosphate bands, corresponding to stretching and bending vibrations, respectively. After adsorption (Fig. 3b), significant spectral changes in the OH^−^, CO_3_^2−^, and PO_4_^3−^ regions indicate surface functionalization and fluoride uptake. The peak at 3200–3600 cm^−1^ notably decreases, suggesting fluorapatite formation due to OH^−^ group replacement on the MRS-HAp surface^15^.

#### XPS results

**Fig. 4:**
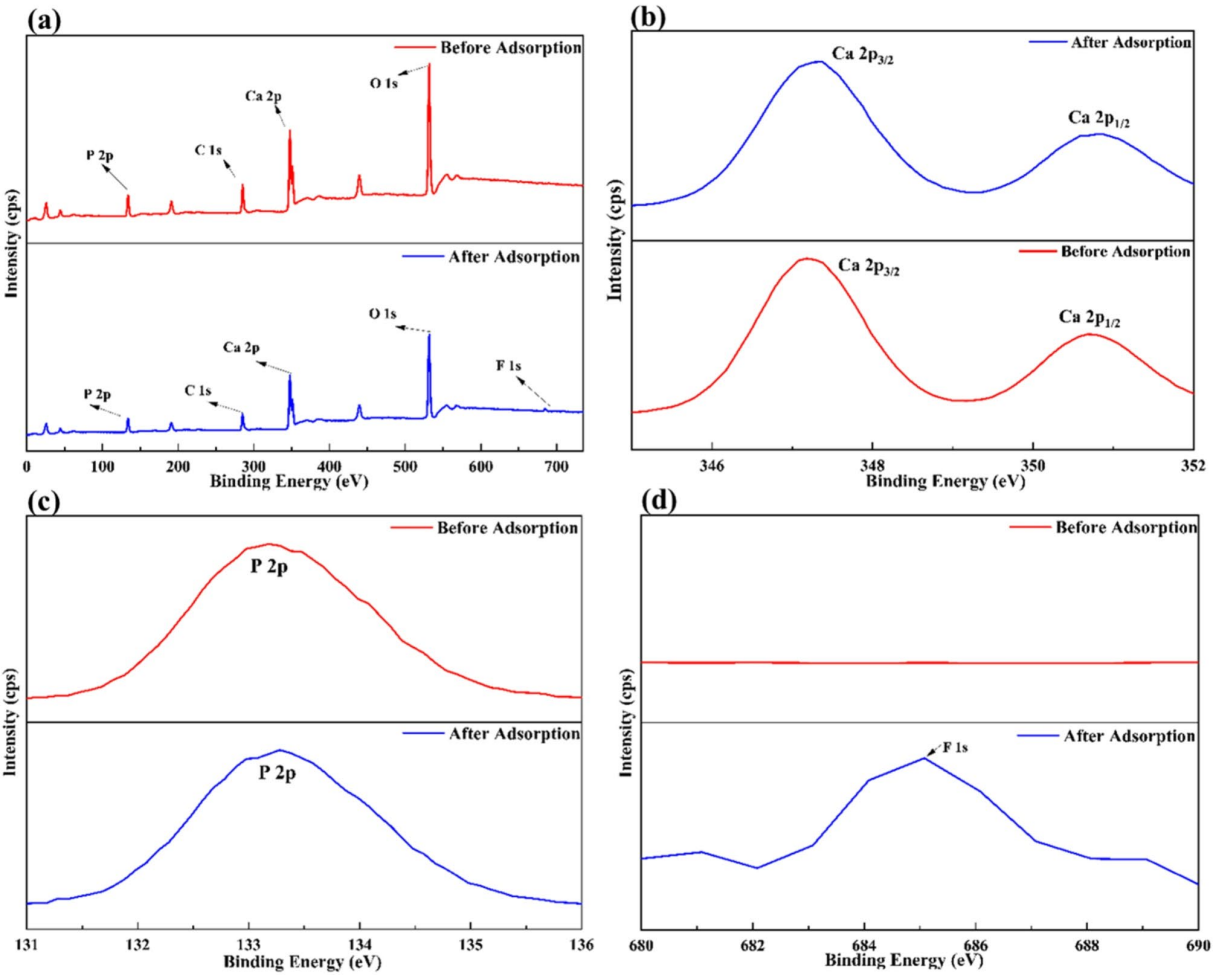
XPS analysis of MRS-HAp (**a**) full spectrum and individual spectra of (**b**) Ca, (**c**) P, and (**d**) F before and after fluoride adsorption.

The XPS analysis of MRS-HAp (Fig. 4a) investigated the chemical states of its elements, revealing binding energy peaks at 133.18 eV (P), 347.18 eV (Ca2P_3/2_), 350.68 eV (Ca2P_1/2_), 285.08 eV (C), and 532.08 eV (O). The existence of P, Ca, O, and C in both spectra confirms their roles as primary surface constituents of hydroxyapatite, a calcium phosphate compound. After adsorption, the XPS analysis shows the binding energy of Ca 2p_3/2_ shifting from 347.18 to 347.38 eV and Ca 2p_1/2_ from 350.68 to 350.78 eV (Fig. 4b), suggesting OH^−^ replacement by fluoride ions. Similarly, the P peak shifts from 133.18 to 133.28 eV due to interactions between OH⁻ and fluoride ions (Fig. 4c). A new peak at 684.58 eV emerges post-adsorption, corresponding to fluoride (Fig. 4d), confirming fluoride incorporation onto the MRS-HAp surface^23^ and supporting the adsorption process, as corroborated by EDS analysis.

### Adsorption results

#### Effect of pH

**Fig. 5:**
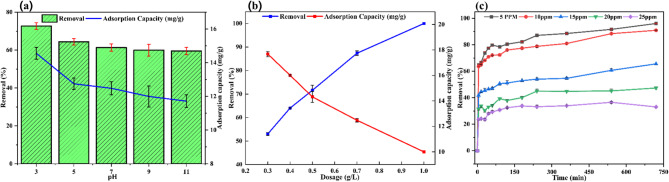
Batch adsorption studies for fluoride removal using MRS-HAp (**a**) effect of pH, (**b**) effect of adsorbent dosage, and (**c**) effect of initial fluoride concentration.

The impact of pH on fluoride removal by MRS-HAp is depicted in Fig. 5a. The highest fluoride removal efficiency, 72.73%, and adsorption capacity, 14.56 mg/g, were observed at pH 3. This high efficiency at pH 3 is attributed to the protonation of surface functional moieties, which imparts MRS-HAp positive charges, enhancing the attraction of negative fluoride ions^22^. Additionally, fluoride ions replace OH^−^ groups in the HAp structure, forming fluorapatite, as confirmed by FTIR studies. The dissolution-reprecipitation process also releases Ca^2+^ ions, which react with fluoride to form insoluble CaF_2_ precipitates^24^. As pH increased from 3 to 11, both removal performance and adsorption capacity gradually decreased, reaching a minimum of 59.52% and 11.72 mg/g at pH 11. At pH 9 and 11, the efficiency of MRS-HAp declines due to competition between fluoride and OH⁻ ions and a reduced surface charge. The maximum fluoride removal in acidic conditions aligns with findings from studies using various adsorbents, such as HAp derived from calcium nitrate tetrahydrate^13^ and *Corbula trigona*^25^.

The pH at the point of zero charge (pH_zpc_) of MRS-HAp was calculated to be 7.22. Below this pH_zpc_, the positively charged adsorbent surface promotes fluoride adsorption via electrostatic attraction and protonation of PO_4_^3−^ and OH^−^ groups^13^. Ion exchange takes place as fluoride ions replace OH⁻ in the HAp lattice, while partial dissolution releases Ca^2+^ ions, forming CaF_2_^24^. Above pH 7.22, the surface becomes negatively charged, causing electrostatic repulsion and heightened competition from OH⁻ ions, which lowers adsorption efficiency. Consequently, pH 3 was chosen for subsequent experiments based on these findings.

#### Effect of dosage

As the dosage of MRS-HAp increases from 0.3 to 1.0 g/L, fluoride removal efficiency increases substantially from 52.59 to 100% (Fig. 5b). However, the adsorption capacity drops concurrently from 17.68 to 10 mg/g. This inverse relationship between removal efficiency and adsorption capacity is attributed to fundamental adsorption mechanisms, where higher dosages enhance removal but reduce the capacity per unit mass of adsorbent.

This trend results from the saturation effect and a reduced fluoride-to-adsorbent ratio. With excess MRS-HAp, competition for available fluoride ions increases among adsorbent particles, leaving some adsorption sites unoccupied and lowering fluoride uptake per gram of MRS-HAp. The optimal dosage was determined to be 0.5 g/L, achieving 71.58% removal and an adsorption capacity of 14.32 mg/g, as indicated by the intersection point in Fig. 5b.

#### Effect of initial concentration and contact time

The effects of fluoride concentration and contact time duration on adsorption by MRS-HAp are depicted in Fig. 5c, revealing an inverse relationship between initial concentration and adsorption efficiency. Removal efficiency dropped from 91.61% at 5 mg/L to 36.53% at 25 mg/L, whereas adsorption capacity rose from 9.61 to 18.27 mg/g. Initially, removal efficiency surged sharply before reaching equilibrium, ranging between 60 and 80% depending on the starting fluoride concentration. Rapid adsorption occurred as fluoride ions occupied available sites, boosting site occupancy. However, at higher concentrations (15, 20, and 25 mg/L), competition for sites intensified as binding spots approached saturation, reducing removal performance compared to lower concentrations.

The adsorption of fluoride onto MRS-HAp exhibits a three-phase kinetic trend. In the first 60 min, rapid adsorption occurs due to abundant active binding sites, enabling quick capture of fluoride ions on the surface, driven primarily by surface adsorption mechanisms^26^. From 60 to 240 min, the adsorption rate slows as sites become occupied, and pore diffusion takes over, with fluoride ions gradually diffusing into internal pores. Between 360 and 540 min, the system reaches equilibrium, showing minimal changes in fluoride concentration as a plateau forms, indicating that most active spots are saturated and adsorption has stabilized^25^. Consequently, 10 mg/L was selected as the optimal fluoride concentration, with an equilibrium time of 540 min.

#### Adsorption isotherms

**Fig. 6:**
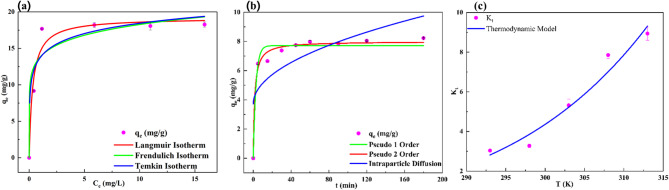
Modeling studies for fluoride adsorption by MRS-HAp (**a**) adsorption isotherms, (**b**) adsorption kinetics, and (**c**) thermodynamic analysis.

Various adsorption isotherms were applied to examine the experimental data, as shown in Fig. 6a, with fitting parameters listed in Table 1. Non-linear fitting was employed to determine the model parameters. The Langmuir model curve in Fig. 6a closely matches the experimental data, evidenced by a high R^2^ = 0.9626, indicating a strong correlation and suggesting that the adsorption process aligns with Langmuir’s assumptions. The low χ^2^ value of 2.65 further confirms this good fit. The Langmuir model implies monolayer adsorption onto uniform surfaces with a definite number of binding spots, supporting the idea that fluoride ions occupy specific adsorption sites on MRS-HAp.

In contrast, the Freundlich model explains multilayer adsorption onto heterogeneous surfaces and does not assume adsorption saturation. Its R^2^ value of 0.9077 indicates a moderate fit, but a higher χ^2^ value of 6.55 suggests it is less accurate for this system. The Temkin model, which accounts for adsorbent-adsorbate interactions and assumes a linear decrease in adsorption heat with coverage, shows significant deviation from the experimental data. With a low R^2^ value of 0.6313, it reflects a poor correlation, and despite a relatively low χ^2^ value of 7.80, it fails to effectively describe the adsorption behavior of MRS-HAp. The maximum adsorption capacity of MRS-HAp was determined as 19.19 mg/g at 303 K, surpassing values from other fluoride adsorption studies using HAp, such as *Corbula trigona* shell-derived HAp at 0.56 mg/g^24^ and Al-derived MOF at 16.53 mg/g^27^. Furthermore, the comparative analysis (Table 2) highlights the superior fluoride adsorption capacity of MRS-HAp. Despite having a moderate surface area (100.42 m^2^/g), MRS-HAp outperforms other reported adsorbents, including bone char (11.98 mg/g)^19^, hydroxyapatite–biomass nanocomposites (10.0 mg/g)^18^, and hydroxyapatite–CMC (10.7 mg/g)^28^. Notably, MRS-HAp also exhibits significantly higher efficiency than rice husk biochar (17.39 mg/g)^29^ and modified *Corbula trigona* shell HAp (4.517 mg/g)^25^, even though the latter has a much higher surface area (249.3 m^2^/g). These results indicate that factors like crystal structure and surface chemistry, beyond the surface area, significantly influence fluoride adsorption, making MRS-HAp a promising low-cost and eco-friendly adsorbent.

**Table 2:** Comparative study of MRS-HAp with different adsorbents for the removal of fluoride.

Sr. no	Adsorbent	Surface area (m^2^/g)	Adsorption conditions					Maximum adsorption Capacity (mg/g)	Reference
			pH	Dose (g/L)	Time (min)	Concentration (mg/L)	Temp (° C)		
1	Modified *Corbula trigona* shell powder HAp	249.3	7.5	5	175	2.2–16.8	25	4.517	^25^
2	Novel hydroxyapatite-biomass nanocomposites	–	5.5	4.0	150	3–80	30	10.0	^18^
3	Hydroxyapatite- carboxymethyl cellulose	90.687	6.9	0.04	30	10–40	30	10.7	^28^
4	Bone char	79.34	7	1	1440	5–50	20	11.98	^19^
5	Rice husk activated biochar	6.12	7	0.2	120	50	30	17.39	^29^
6	*Marcia recens* shells HAp	100.42	3	0.5	720	10	30	19.19	This study

#### Adsorption kinetics

The PFO model yielded a calculated q_e_ of 7.71 mg/g, below the experimental q_e_ of 8.23 mg/g, indicating rapid initial adsorption. Its high R^2^ (0.9697) and low χ^2^ (0.23) suggest a good fit (Table 1). The PSO model provided a q_e_ (8 mg/g) closely aligned with experimental data, offering a superior fit with a higher R^2^ (0.9873) and lower χ^2^ (0.09), indicating that chemisorption drives the process. The IPD model portrayed that pore diffusion occurs but is not the sole rate-limiting step; its low R^2^ (0.5508) and high χ^2^ (3.43) confirm a poor fit, highlighting the dominance of surface adsorption and boundary layer effects. Among the models, PSO best describes the kinetics (Fig. 6b), consistent with studies on coffee bean biochar^30^, and Al-modified HAp^31^.

#### Thermodynamic investigation

The impact of temperature on fluoride adsorption by MRS-HAp was studied using the non-linear Van’t Hoff equation, with results shown in Fig. 6c; Table 1. A high R^2^ of 0.9556 and a low χ^2^ of 0.42 indicate a strong relation between the experimental data and the model. Negative ΔG° values across all tested temperatures (293 K to 313 K) confirm that adsorption is spontaneous and feasible^32^. The positive ΔH° (45.5791 kJ/mol) reveals an endothermic process, with efficiency increasing at higher temperatures, and its value above 40 kJ/mol classifies it as chemisorption. A positive ΔS° suggests increased randomness at the solid-liquid interface, likely due to fluoride ions shedding their solvation sheath and MRS-HAp surface sites restructuring during adsorption^22^. These results establish fluoride adsorption on MRS-HAp as a spontaneous, endothermic, chemisorption-driven process, consistent with findings from studies using chemical-based hydrothermal HAp^29^, and Al-MOF adsorbents^27^.

#### Plausible mechanism of fluoride adsorption

**Fig. 7:**
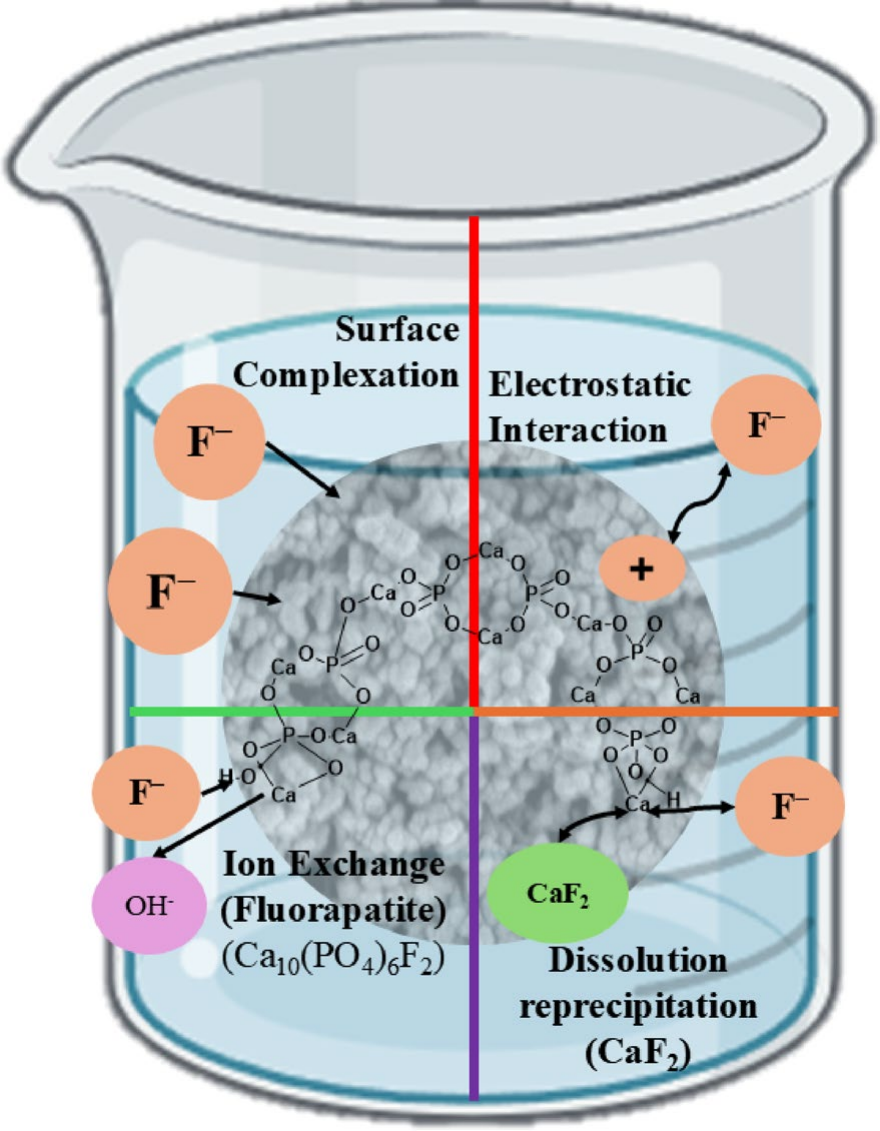
Plausible mechanism of fluoride removal onto MRS-HAp.

The mechanisms governing fluoride adsorption onto MRS-HAp were investigated using a combination of characterization techniques and batch adsorption experiments, as depicted in Fig. 7. The primary mechanism identified is chemisorption, which is facilitated by multiple processes, including ion exchange, electrostatic interactions, dissolution-reprecipitation, and surface complexation. Among these, ion exchange plays a critical role, whereby fluoride ions substitute for OH^−^ within the HAp lattice. This substitution was evidenced by FTIR spectroscopy, which revealed a notable reduction in OH^−^ absorption bands (3200–3600 cm^− 1^) following adsorption, consistent with the formation of fluorapatite^33^. The efficiency of this ion exchange process is maximized under acidic conditions, where a low pH promotes protonation of PO_4_^3−^ and OH^−^ groups on the HAp surface. This protonation enhances the positive surface charge, thereby strengthening the electrostatic attraction and facilitating fluoride uptake^34^.

The pH_zpc_ of MRS-HAp, determined to be 7.22, indicates that the material carries a positive surface charge at pH values below this threshold, thereby promoting fluoride adsorption, whereas adsorption efficiency diminishes at higher pH levels^15^. At pH 3, the release of Ca^2+^ ions was observed, leading to the formation of CaF_2_, as confirmed by XPS with a characteristic peak at 684.58 eV. This metal-fluoride formation was further corroborated by fluoride detection through EDS analysis^33^. Adsorption isotherm studies exhibited that the Langmuir model best describes the data, suggesting a monolayer adsorption process. Kinetic investigations revealed an initial rapid adsorption phase followed by a slower pore diffusion stage, with the PSO model providing a superior fit, indicative of chemisorption. The thermodynamic analysis yielded a positive enthalpy change (ΔH° > 0), reinforcing the endothermic and spontaneous nature of the adsorption process^31^. Similar mechanisms have been documented in related studies, including those involving hydroxyapatite derived from mussel shells^15^, EDTA-modified hydroxyapatite^17^, and chemically synthesized hydrothermal hydroxyapatite^31^.

#### Simulated fluoride water studies

**Fig. 8:**
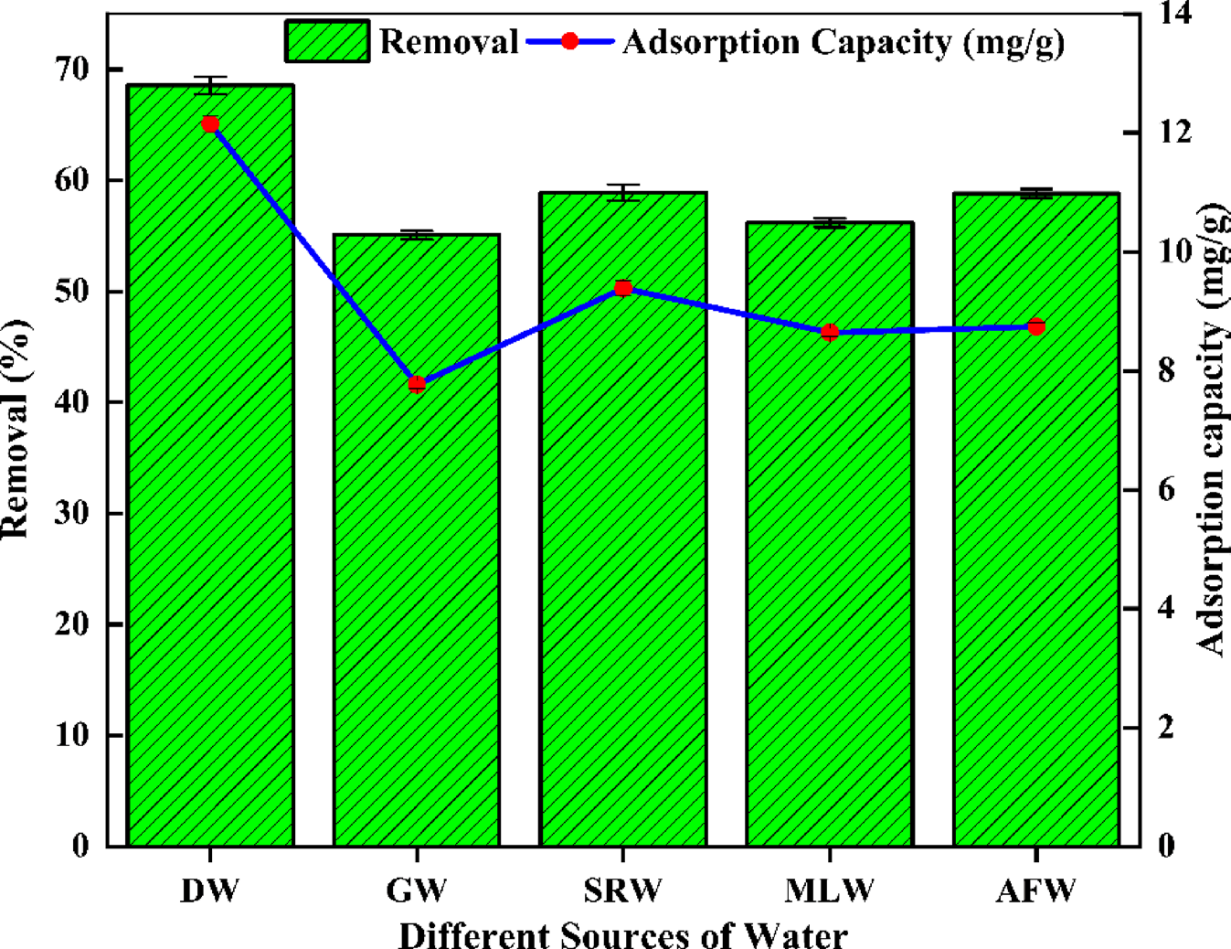
Fluoride removal performance of MRS-HAp in spiked water samples from different water sources.

Simulated fluoride wastewater studies were conducted by preparing fluoride stock solutions in real water samples and evaluating performance under optimized conditions. The results, illustrated in Fig. 8, revealed fluoride removal efficiencies of 55.08% for GW, 58.88% for SRW, 56.17% for MLW, 58.82% for AFW, and 68.55% for distilled water (DW) control. The corresponding adsorption capacities were determined to be 12.15 mg/g, 7.77 mg/g, 9.39 mg/g, 8.64 mg/g, and 8.74 mg/g, respectively. These variations in performance are attributed to the influence of pre-existing minerals and ions within the natural water matrices, which differ from the controlled conditions of DW. These findings highlight the effectiveness of MRS-HAp for fluoride removal, demonstrating its applicability not only in idealized aqueous systems but also in complex, real-world water environments.

#### Regeneration and reusability studies of MRS-HAp

**Fig. 9:**
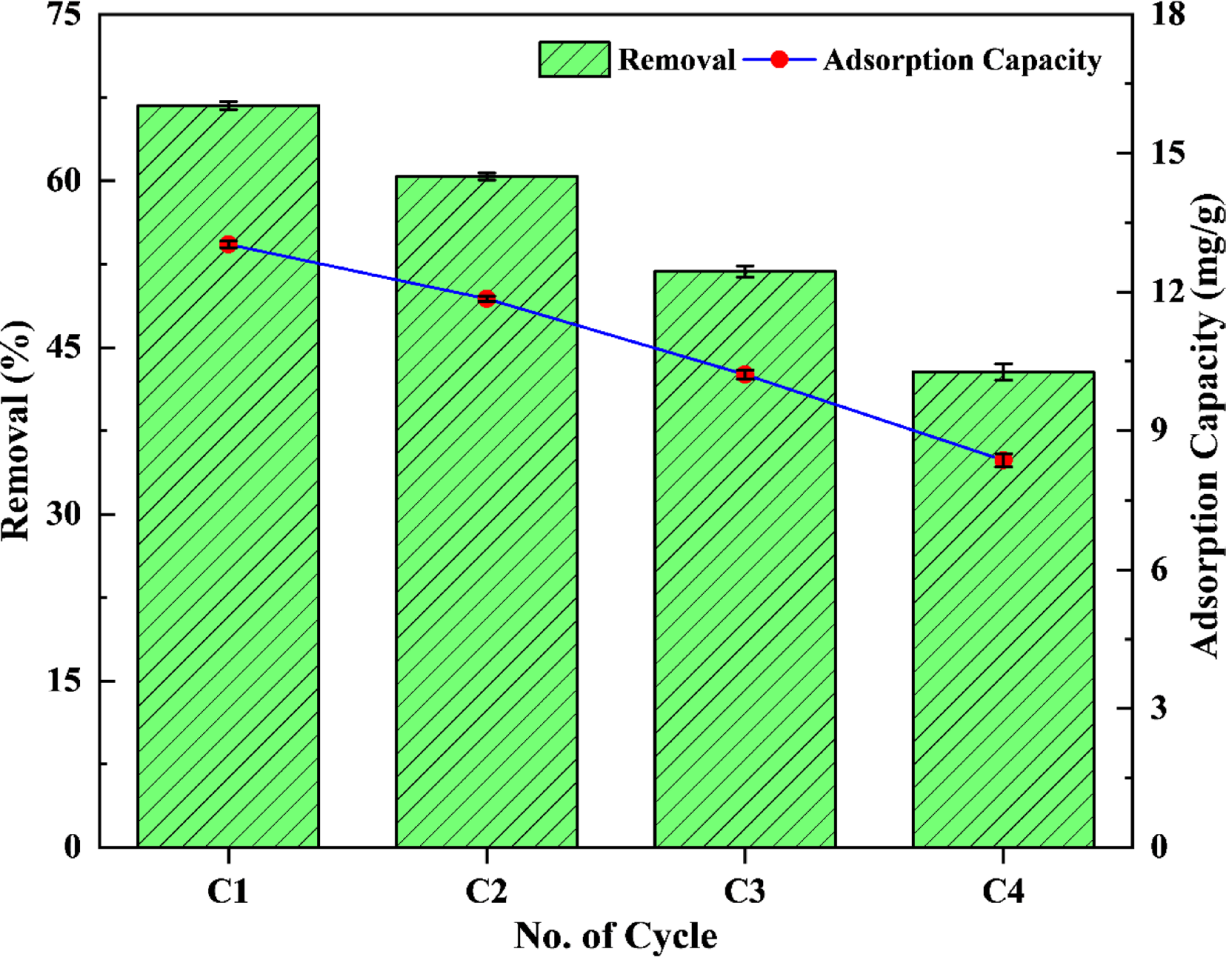
Regeneration and reusability studies of MRS-HAp for fluoride removal.

Figure 9 illustrates the fluoride removal efficiency and adsorption capacity across four regeneration cycles. The removal efficiency declined from 66.80 to 42.78%, while the corresponding adsorption capacity decreased from 13.03 to 8.36 mg/g. This reduction may be attributed to the blockage of active sites, likely caused by the replacement of OH⁻ with fluoride ions^35^, as well as alterations to surface functionalities within the MRS-HAp crystal lattice^36^. This decline in the removal efficiency has been documented in related studies, including those with hydroxyapatite derived from *Anadara granosa*^35^, nitric acid-activated alumina^14^, and Mg/Al-based LDH powder^37^.

## Conclusion

In this study, hydroxyapatite derived from *Marcia recens* shells (MRS-HAp) was successfully synthesized and its efficacy in fluoride removal from water was thoroughly assessed. The material’s morphology and crystallinity were characterized using FESEM and XRD, while FTIR and XPS elucidated its functional groups and chemical composition. BET analysis further confirmed the mesoporous structure of MRS-HAp, supporting its suitability for adsorption. Batch adsorption experiments demonstrated a strong pH dependence, with optimal fluoride removal occurring at pH 3, attributed to enhanced electrostatic interactions. Adsorption adhered to a pseudo-second-order kinetic model, indicative of chemisorption, and conformed to the Langmuir isotherm, suggesting monolayer adsorption with a capacity of 19.19 mg/g. Thermodynamic analysis substantiated that the process was spontaneous, feasible, and endothermic. The adsorption mechanism involved ion exchange, electrostatic interactions, and surface complexation, as corroborated by characterization studies. Tests with spiked water samples demonstrated substantial fluoride removal efficiencies, affirming the material’s efficacy across complex water matrices. Despite a gradual decline in performance over successive cycles, MRS-HAp exhibited acceptable reusability for practical fluoride remediation applications. Collectively, these findings establish MRS-HAp as a promising, cost-effective, and sustainable adsorbent for fluoride removal.

## Data Availability

The authors declare that the dataset supporting the results of this investigation are presented within the paper itself. Raja Selvaraj may be contacted to get the data used for this investigation.

## Declarations

### Guidelines

The authors affirm that the use of shells in this report complies with international, national, and/or institutional guidelines.

### Permission to collect the shells

The shells used in this study were identified and authenticated by Dr. A Biju Kumar, Senior Professor and Head, Department of Aquatic Biology and Fisheries, University of Kerala, Kerala India.

### Generative AI and AI-assisted technologies in the writing process

The authors utilized ChatGPT to enhance grammar and refine sentence structure to prepare this article. Following its use, they thoroughly reviewed and edited the contents as necessary, taking full accountability for the final publication.

## References

[1] Garg, A. Emerging contaminants in subsurface: Sources, remediation, and challenges, in *Advances in Remediation Techniques for Polluted Soils and Groundwater*, 233–257. 10.1016/B978-0-12-823830-1.00014-6 (2021).

[2] Shit, P. K. et al. *Geospatial Practices in Natural Resources Management*. 10.1007/978-3-031-38004-4 (2024).

[3] Narsimha, A. & Sudarshan, V. Drinking water pollution with respective of fluoride in the semi-arid region of basara, nirmal district, Telangana state, India. *Data Br.* **16**, 752–757. 10.1016/j.dib.2017.11.087 (2018). 10.1016/j.dib.2017.11.087 PMC5735257 29270457

[4] Ghosh, B., Begum, S. N. & Hossain, M. A review on source, impacts and mitigation measures of groundwater fluoride contamination: A major health issue. *Int. J. Adv. Res. Trends Sci.* **1** (1), 34–40. 10.70035/ijarts.2022.1134-40 (2022).

[5] Gebrewold, B. D. et al. Low cost materials for fluoride removal from groundwater. *J. Environ. Manag.* **370**. 10.1016/j.jenvman.2024.122937 (2024). 10.1016/j.jenvman.2024.122937 39490019

[6] Toghan, A. et al. Effect of Adsorption and interactions of new triazole-thione-schiff bases on the corrosion rate of carbon steel in 1 M HCl solution: Theoretical and experimental evaluation. *ACS Omega* **9**(6), 6761–6772. (2024). 10.1021/acsomega.3c08127 10.1021/acsomega.3c08127 PMC10870402 38371797

[7] de Carvalho Costa, L. R. et al. Exploring key parameters in adsorption for effective fluoride removal: A comprehensive review and engineering implications. *Appl. Sci.* **14** (5). 10.3390/app14052161 (2024).

[8] Wei, Y., Wang, L., Li, H., Yan, W. & Feng, J. Synergistic fluoride adsorption by composite adsorbents synthesized from different types of materials—A review. *Front. Chem.* **10**. 10.3389/fchem.2022.900660 (2022). 10.3389/fchem.2022.900660 PMC9114667 35601557

[9] Arokiasamy, P. et al. Synthesis methods of hydroxyapatite from natural sources: A review. *Ceram. Int.* **48** (11), 14959–14979. 10.1016/j.ceramint.2022.03.064 (2022).

[10] Li, C., Li, X., Zhang, Q., Li, L. & Wang, S. The alkaline fusion-hydrothermal synthesis of hydroxyapatite-zeolite (HAP-ZE) from blast furnace slag (BFS): Effects of reaction temperature. *Minerals* **11** (11). 10.3390/min11111160 (2021).

[11] Krishani, M., Suhaimi, H. & Sambudi, N. S. A review of hydroxyapatite: Sustainable product development in terms of waste valorization, in *What to Know about Hydroxyapatite*, 219–243. (2023).

[12] Standard, I. Methods of Sampling and Test (Physical and Chemical) for Water and Waste Water Is: 3025. (Bureau of Indian Standards, New Delhi, India, 1998).

[13] Sekar, S. et al. Enhanced F– adsorption and regeneration efficiency of pectin anchored on hydroxyapatite (HAp/PEC) nanocomposites. *J. Environ. Chem. Eng.* **12** (5). 10.1016/j.jece.2024.113738 (2024).

[14] Kumari, U., Behera, S. K., Siddiqi, H. & Meikap, B. C. Facile method to synthesize efficient adsorbent from alumina by nitric acid activation: Batch scale defluoridation, kinetics, isotherm studies and implementation on industrial wastewater treatment. *J. Hazard. Mater.* **381**(July 2019), 120917. 10.1016/j.jhazmat.2019.120917 (2020). 10.1016/j.jhazmat.2019.120917 31376661

[15] Zou, Y. et al. Preparation of hydroxyapatite and its elimination of excess fluoride from aqueous solution. *RSC Adv.* **14** (36), 26103–26114. 10.1039/D4RA02147A (2024). 39161437 10.1039/d4ra02147a PMC11332186

[16] Gomes, G. C. et al. Rapid stoichiometric analysis of calcium–phosphorus ratio on hydroxyapatite targets by one-point calibration laser-induced breakdown spectroscopy (OPC-LIBS). *Spectrochim. Acta - Part B At. Spectrosc.* **184**(2021). 10.1016/j.sab.2021.106250 (2020).

[17] Huang, S. et al. Enhanced water defluoridation using ion channel modified hydroxyapatite: Experimental, mechanisms and DFT calculation. *Appl. Surf. Sci.* **615**. 10.1016/j.apsusc.2023.156351 (2023).

[18] Scheverin, V. N., Horst, M. F. & Lassalle, V. L. Novel hydroxyapatite-biomass nanocomposites for fluoride adsorption. 10.1016/j.rineng.2022.100648 (2022). 10.1007/s10653-024-01981-w 38695943

[19] Shahid, M. K., Kim, J. Y. & Choi, Y. G. Synthesis of bone char from cattle bones and its application for fluoride removal from the contaminated water. *Groundw. Sustain. Dev.* **8**(December 2018), 324–331. 10.1016/j.gsd.2018.12.003 (2019).

[20] Safari-Gezaz, M. & Parhizkar, M. Effect of ionic liquid as a surfactant in hydroxyapatite coatings for improvement corrosion resistance of Ti-6Al-4V substrates for implant applications. *Heliyon* **10** (24), e40990. 10.1016/j.heliyon.2024.e40990 (2024). 39720066 10.1016/j.heliyon.2024.e40990 PMC11665465

[21] Uysal, I., Severcan, F. & Evis, Z. Characterization by fourier transform infrared spectroscopy of hydroxyapatite co-doped with zinc and fluoride. *Ceram. Int.* **39**, 7727–7733. 10.1016/j.ceramint.2013.03.029 (2013).

[22] Nayak, B., Samant, A., Patel, R. & Misra, P. K. Comprehensive Understanding of the kinetics and mechanism of fluoride removal over a potent nanocrystalline hydroxyapatite surface. *ACS Omega* **2** (11), 8118–8128. 10.1021/acsomega.7b00370 (2017). 31457358 10.1021/acsomega.7b00370 PMC6645433

[23] Lee, J. et al. Tuning two interfaces with fluoroethylene carbonate electrolytes for High-Performance li/lco batteries. *ACS Omega*. **4** (2), 3220–3227. 10.1021/acsomega.8b03022 (2019). 31459539 10.1021/acsomega.8b03022 PMC6648377

[24] Yapo, N. S. et al. Bivalve shells (*Corbula trigona*) as a new adsorbent for the defluoridation of groundwater by adsorption-precipitation. *J. Environ. Sci. Heal - Part. Toxic/Hazardous Subst. Environ. Eng.* **56** (6), 694–704. 10.1080/10934529.2021.1917937 (2021). 10.1080/10934529.2021.1917937 33985405

[25] Yapo, N. S. et al. Removal of fluoride in groundwater by adsorption using hydroxyapatite modified *Corbula trigona* shell powder. *Chem. Eng. J. Adv.* **12**. 10.1016/j.ceja.2022.100386 (2022).

[26] Ayalew, A. A. Comparative adsorptive performance of adsorbents developed from kaolin clay and limestone for de-fluoridation of groundwater, *South African J. Chem. Eng.* **44**(October 2022), 1–13. 10.1016/j.sajce.2022.11.002 (2023).

[27] Wang, Z. et al. New easily recycled carrier based polyurethane foam by loading Al-MOF and Biochar for selective removal of fluoride ion from aqueous solutions. *Sci. Total Environ.* **901**. 10.1016/j.scitotenv.2023.166312 (2023). 10.1016/j.scitotenv.2023.166312 37586503

[28] Fernando, M. S. et al. Biopolymer-based nanohydroxyapatite composites for the removal of fluoride, lead, cadmium, and arsenic from water. *ACS Omega* **6** (12), 8517–8530. 10.1021/acsomega.1c00316 (2021). 33817513 10.1021/acsomega.1c00316 PMC8015138

[29] Kumar, R. et al. Rice husk biochar - A novel engineered bio-based material for transforming groundwater-mediated fluoride cycling in natural environments. *J. Environ. Manag.* **343**. 10.1016/j.jenvman.2023.118222 (2023). 10.1016/j.jenvman.2023.118222 37235991

[30] dos Santos, H. V. R., Cuba, R. M. F., Scalize, P. S. & Teran, F. J. C. Fluoride adsorption by Coffee-Ground Biochar functionalized with hydrogen peroxide. *J. Ecol. Eng.* **25** (12), 244–260. 10.12911/22998993/194494 (2024).

[31] Ren, M., Jia, X., Huang, T., Wu, B. & Yang, J. Fluoride removal by hydroxyapatite modi Fi ed with anhydrous aluminum chloride. **24**(6), 2172–2184. 10.2166/ws.2024.117 (2024).

[32] Tran, H. N. Improper Estimation of thermodynamic parameters in adsorption studies with distribution coefficient K D(q e/ C e) or Freundlich Constant (K F): Considerations from the derivation of dimensionless thermodynamic equilibrium constant and suggestions. *Adsorpt. Sci. Technol.* **2022**10.1155/2022/5553212 (2022).

[33] Sundaram, C. S., Viswanathan, N. & Meenakshi, S. Defluoridation chemistry of synthetic hydroxyapatite at nano scale: Equilibrium and kinetic studies, *J. Hazard. Mater.*, vol. 155, no. 1–2, pp. 206–215, Jun. (2008). 10.1016/j.jhazmat.2007.11.048 10.1016/j.jhazmat.2007.11.048 18162304

[34] Rathnayake, A. et al. Essence of hydroxyapatite in defluoridation of drinking water: A review. *Environ. Pollut.* **311**(December 2021 ), 119882. 10.1016/j.envpol.2022.119882 (2022). 10.1016/j.envpol.2022.119882 35934148

[35] Mtavangu, S. G., Mahene, W., Machunda, R. L., van der Bruggen, B. & Njau, K. N. Cockle (*Anadara granosa*) shells-based hydroxyapatite and its potential for defluoridation of drinking water. *Results Eng.* **13**, 100379. 10.1016/j.rineng.2022.100379 (2022).

[36] González-Ponce, H. A., Mendoza-Castillo, D. I., Bonilla-Petriciolet, A., Reynel-Ávila, H. E. & Camacho-Aguilar, K. I. Regeneration analysis of bone char used in water defluoridation: Chemical desorption route, surface chemistry analysis and modeling. *Int. J. Chem. Eng.* **2023**. 10.1155/2023/8378162 (2023).

[37] Han, M. et al. Mg/Al-LDH-Cl- for fluoride removal from water: High-capacity adsorption and recyclable regeneration. *Ind. Eng. Chem. Res.*10.1021/acs.iecr.5c00840 (2025).

